# Supplementary material for: The Nucleocapsid (N) Proteins of Different Human Coronaviruses Demonstrate a Variable Capacity to Induce the Formation of Cytoplasmic Condensates
Source: Int J Mol Sci. 2024 Dec 7;25(23):13162. doi: 10.3390/ijms252313162 (PMC11642284; doi:10.3390/ijms252313162)
Supplement: Supplementary file 1 [file ijms-25-13162-s001.zip › ijms-3352025-supplementary.pdf]

The nucleocapsid (N) proteins of different human coronaviruses demonstrate a variable capacity to induce the formation of cytoplasmic condensates

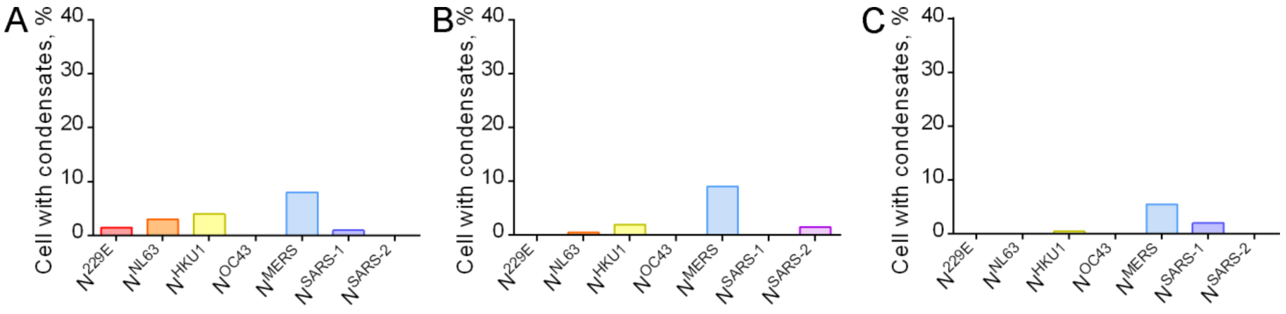

Supplementary Figure. The percentage of cells with cytoplasmic condensates among all cells expressing N proteins of different HCoVs 24 hours after transfection. (**A**) HT1080 cells, (**B**) U2OS cells, and (**C**) A549 cells. Data for HeLa cells are shown in Figure 1C and Supplementary Figure.
